# HDL Cholesterol and the Triglyceride-to-HDL Cholesterol Ratio in Relation to Coronary Artery Disease Complexity in Patients with Type 2 Diabetes Mellitus

**DOI:** 10.3390/jcm15166400

**Published:** 2026-08-19

**Authors:** Mehmet Aydoğan, Mustafa Lütfi Yavuz, Emre Yalçın, Pelin Karaca Özer, Samim Emet

**Affiliations:** Department of Cardiology, Istanbul Faculty of Medicine, Istanbul University, 34093 Istanbul, Türkiye; mustafayavuz135@gmail.com (M.L.Y.); emreyalcin@istanbul.edu.tr (E.Y.); pkaracaozer@gmail.com (P.K.Ö.); samim03@hotmail.com (S.E.)

**Keywords:** type 2 diabetes mellitus, coronary artery disease, HDL cholesterol, SYNTAX score, coronary angiography, dyslipidemia

## Abstract

**Background/Objectives**: Patients with type 2 diabetes mellitus (T2DM) frequently develop diffuse and anatomically complex coronary artery disease (CAD), resulting in a substantially increased cardiovascular risk. Although both HDL-C and the triglyceride-to-high-density lipoprotein cholesterol (TG/HDL-C) ratio have been associated with cardiovascular risk, their relative associations with and discriminatory performance for angiographic CAD complexity remain uncertain. This study aimed to evaluate and compare these two lipid parameters in relation to CAD complexity as assessed by the SYNTAX score. **Methods**: A retrospective, observational, single-center study was conducted involving 167 consecutive T2DM patients who underwent diagnostic coronary angiography. CAD complexity was quantified using the SYNTAX score, with patients categorized into low (≤22) and higher (>22) complexity groups. Statistical evaluation included correlation analyses, univariable and multivariable logistic regression, and receiver operating characteristic (ROC) curve analysis. **Results**: Of the 167 patients, 48 (28.7%) were classified with higher SYNTAX scores. HDL-C showed a significant inverse correlation with the SYNTAX score (r = −0.339, *p* < 0.001), whereas the TG/HDL-C ratio showed a weak positive correlation (r = 0.159, *p* = 0.040). In separate multivariable models adjusted for sex, both HDL-C (OR 0.944, 95% CI 0.908–0.980; *p* = 0.003) and the TG/HDL-C ratio (OR 1.085, 95% CI 1.003–1.175; *p* = 0.041) remained associated with a higher SYNTAX score. HDL-C showed a numerically higher AUC than the TG/HDL-C ratio (0.659 vs. 0.621); however, the difference between the AUCs was not statistically significant (DeLong *p* = 0.268). **Conclusions**: Lower HDL-C and a higher TG/HDL-C ratio remained associated with a higher SYNTAX score after adjustment for sex. Although HDL-C showed numerically better discrimination, no statistically significant difference in discriminatory performance was demonstrated between the two markers.

## 1. Introduction

Coronary artery disease (CAD) remains the primary cause of morbidity and mortality in patients with type 2 diabetes mellitus (T2DM). Individuals with T2DM face a two- to four-fold higher risk of developing CAD compared with the general population and frequently present with diffuse, multivessel, and anatomically complex coronary lesions [1,2]. Diabetic dyslipidemia is characterized by reduced high-density lipoprotein cholesterol (HDL-C), elevated triglycerides, and an increased proportion of small dense low-density lipoprotein particles. Despite the success of LDL-C reduction as a cornerstone of lipid-lowering therapy, substantial residual cardiovascular risk persists even among patients receiving contemporary treatment, suggesting that additional lipid-related biomarkers may improve cardiovascular risk stratification in this high-risk population. Patients with T2DM therefore represent a particularly relevant population for evaluating HDL-related lipid markers because they commonly exhibit both atherogenic dyslipidemia and diffuse, anatomically complex CAD. Focusing specifically on this high-risk group may help clarify the relationship between routinely available lipid parameters and coronary anatomical complexity [2,3,4].

HDL-C has attracted considerable attention because its protective role extends beyond reverse cholesterol transport. HDL particles exert multiple anti-atherogenic effects, including antioxidant, anti-inflammatory, antithrombotic, and endothelial-protective activities, while promoting cholesterol efflux from macrophages and preserving endothelial function. However, in T2DM, chronic hyperglycemia, insulin resistance, and oxidative stress promote glycation and oxidation of HDL particles, leading to impaired HDL functionality despite apparently preserved circulating HDL-C concentrations. Consequently, reduced HDL-C may represent not only a quantitative lipid abnormality but also a marker of dysfunctional HDL and advanced coronary atherosclerosis [2,4,5].

The triglyceride-to-high-density lipoprotein cholesterol (TG/HDL-C) ratio has been proposed as a simple surrogate marker of insulin resistance and atherogenic dyslipidemia [5,6]. Elevated TG/HDL-C ratios have been associated with metabolic syndrome, visceral obesity, endothelial dysfunction, and increased cardiovascular risk in several epidemiological studies. Nevertheless, its association with anatomical CAD complexity has shown inconsistent results, particularly after adjustment for conventional cardiovascular risk factors. These discrepancies may reflect differences in study populations, diabetes duration, background lipid-lowering therapy, and clinical endpoints [6,7].

The SYNTAX score is a validated angiographic tool for quantifying the anatomical complexity of CAD and has consistently been associated with procedural complexity and adverse cardiovascular outcomes following coronary revascularization [8,9]. Although both HDL-C and the TG/HDL-C ratio have been associated with cardiovascular risk, evidence directly comparing their associations with and discriminatory performance for angiographic CAD complexity specifically in patients with T2DM remains limited. Clarifying their relative discriminatory performance may improve risk stratification using routinely available laboratory parameters. Therefore, the present study aimed to evaluate and compare the associations and discriminatory performance of HDL-C and the TG/HDL-C ratio in relation to angiographic CAD complexity, as assessed by the SYNTAX score, in patients with T2DM.

## 2. Materials and Methods

### 2.1. Study Design and Population

This retrospective, observational, single-center study included 167 consecutive patients with established type 2 diabetes mellitus (T2DM) who underwent elective diagnostic coronary angiography at the Department of Cardiology, Istanbul University, Istanbul Medical Faculty, between April 2021 and September 2022. Clinical, laboratory, and angiographic data were retrospectively retrieved from the institutional electronic medical records. Patients were subsequently classified into two groups according to their SYNTAX score as low (≤22) and higher (>22) for comparative analyses.

T2DM was defined according to the American Diabetes Association criteria or by the documented use of glucose-lowering medications [10]. Patients with type 1 diabetes mellitus, acute coronary syndrome requiring emergency coronary angiography, previous coronary artery bypass graft surgery, significant valvular heart disease, severe hepatic disease, or advanced renal disease were excluded from the study.

Information on background lipid-lowering and concomitant cardiometabolic therapies was incompletely documented in the retrospective dataset. Therefore, specific treatment regimens and treatment intensity could not be reliably characterized.

The study protocol was approved by the Institutional Review Board of Istanbul University, Istanbul Medical Faculty (Approval No. 2022/1705) and was conducted in accordance with the ethical principles outlined in the Declaration of Helsinki [11].

### 2.2. Clinical and Laboratory Assessment

Hypertension was defined as a previous diagnosis of hypertension, current antihypertensive treatment, or repeated office blood pressure measurements ≥140/90 mmHg according to the current European Society of Cardiology guidelines [12]. Venous blood samples were obtained after an overnight fast on the morning of coronary angiography and analyzed using standard automated laboratory methods in the institutional biochemistry laboratory. Routine laboratory parameters, including lipid profile, serum creatinine, hemoglobin, and glycated hemoglobin (HbA1c), were recorded from the hospital database. The triglyceride-to-high-density lipoprotein cholesterol (TG/HDL-C) ratio was calculated by dividing the serum triglyceride concentration by the HDL-C concentration [6].

### 2.3. Angiographic Evaluation and SYNTAX Score Assessment

Coronary angiography was performed using the standard Judkins technique [13]. Significant coronary artery disease (CAD) was defined as the presence of ≥50% luminal diameter stenosis in at least one major epicardial coronary artery [14]. Coronary lesion complexity was assessed using the SYNTAX score, a validated angiographic scoring system that incorporates lesion location, morphology, bifurcation involvement, total occlusion, calcification, tortuosity, lesion length, and thrombus burden to quantify the anatomical complexity of CAD [8,9]. Based on the calculated SYNTAX score, patients were categorized into a low SYNTAX score group (≤22) and a higher SYNTAX score group (>22). This threshold was selected based on the conventional SYNTAX score classification, in which scores ≤ 22 represent low anatomical complexity, whereas scores > 22 represent intermediate-to-high anatomical complexity [9,14].

### 2.4. Statistical Analysis

Statistical analyses were performed using IBM SPSS Statistics software (version 27; IBM Corp., Armonk, NY, USA). The distribution of continuous variables was assessed using the Kolmogorov–Smirnov test. Normally distributed variables are presented as mean ± standard deviation (SD) and were compared using the independent-samples Student’s *t* test, whereas non-normally distributed variables are presented as median (interquartile range [IQR]) and were compared using the Mann–Whitney U test. Categorical variables are expressed as frequencies (percentages) and were compared using the chi-square test or Fisher’s exact test, as appropriate. Correlations between continuous variables were evaluated using Spearman’s rank correlation analysis.

Univariable logistic regression analysis was performed to identify variables associated with a higher SYNTAX score (SYNTAX score > 22). Variables with a *p* value < 0.05 in univariable analysis were entered into the multivariable model. Sex was additionally included irrespective of univariable significance to account for established sex-related differences in HDL-C levels. Given that HDL-C is mathematically incorporated into the TG/HDL-C ratio, multicollinearity between these variables was formally assessed using variance inflation factors (VIFs) and tolerance values. To avoid potential model dependence arising from their simultaneous inclusion, HDL-C and the TG/HDL-C ratio were subsequently evaluated in separate multivariable logistic regression models, with sex included as a covariate in each model. Odds ratios (ORs) are presented with 95% confidence intervals (CIs). Receiver operating characteristic (ROC) curve analysis was performed to evaluate the discriminatory performance of HDL-C and the TG/HDL-C ratio for identifying a higher SYNTAX score, and the optimal cut-off values were determined using the Youden index [15]. The AUCs of HDL-C and the TG/HDL-C ratio were formally compared using the DeLong test, which was performed using Python (version 3.13.5) with an implementation of DeLong’s method [16]. A two-sided *p* value < 0.05 was considered statistically significant.

## 3. Results

### 3.1. Baseline Characteristics

A total of 167 patients with T2DM were included in the study. Based on the SYNTAX score, 119 (71.3%) patients were classified into the low SYNTAX score group (≤22), whereas 48 (28.7%) were classified into the higher SYNTAX score group (>22). The baseline demographic, clinical, and laboratory characteristics of the study population are summarized in Table 1.

The two groups were comparable with respect to age, sex distribution, body mass index, body surface area, hypertension, smoking status, atrial fibrillation, chronic kidney disease, cerebrovascular disease, family history of cardiovascular disease, hemoglobin A1c, serum creatinine, hemoglobin, LDL cholesterol, total cholesterol, and triglyceride concentrations (all *p* > 0.05).

In contrast, patients with higher SYNTAX scores had significantly lower HDL-C concentrations than those with low SYNTAX scores (36.44 ± 8.7 vs. 42.88 ± 11.8 mg/dL, *p* = 0.001). Conversely, the TG/HDL-C ratio was significantly higher in the higher SYNTAX group than in the low SYNTAX group (6.27 ± 5.8 vs. 4.63 ± 3.5, *p* = 0.014).

Overall, HDL-C levels were significantly higher in women than in men (45.06 ± 11.90 vs. 38.37 ± 10.22 mg/dL, *p* < 0.001). When analyzed according to sex, HDL-C levels remained significantly lower in the higher SYNTAX score group in both women (39.47 ± 9.53 vs. 46.74 ± 12.10 mg/dL, *p* = 0.037) and men (35.06 ± 8.08 vs. 40.00 ± 10.81 mg/dL, *p* = 0.022).

When the TG/HDL-C ratio was analyzed according to sex, values were numerically higher in the higher SYNTAX score group in both women (6.81 ± 5.68 vs. 4.58 ± 3.39, *p* = 0.059) and men (6.02 ± 5.95 vs. 4.67 ± 3.67, *p* = 0.100), although these differences did not reach statistical significance.

### 3.2. Correlation Analysis

Correlation analysis demonstrated a significant inverse association between HDL-C and the SYNTAX score (r = −0.339, *p* < 0.001), indicating that lower HDL-C levels were associated with greater coronary artery disease complexity. In contrast, the TG/HDL-C ratio showed a weak but statistically significant positive correlation with the SYNTAX score (r = 0.159, *p* = 0.040).

LDL cholesterol (r = −0.145, *p* = 0.063) and body surface area (r = 0.150, *p* = 0.053) demonstrated correlations that did not reach statistical significance. Similarly, triglycerides, age, body mass index, hemoglobin A1c, serum creatinine, and hemoglobin were not significantly correlated with the SYNTAX score (all *p* > 0.05) (Table 2).

### 3.3. Regression Modeling

Univariable logistic regression analysis identified both HDL-C (OR 0.942, 95% CI 0.907–0.977; *p* = 0.001) and the TG/HDL-C ratio (OR 1.084, 95% CI 1.003–1.171; *p* = 0.042) as significantly associated with a higher SYNTAX score. No relevant multicollinearity was detected between HDL-C and the TG/HDL-C ratio (VIFs < 2). Given the mathematical incorporation of HDL-C into the TG/HDL-C ratio, the two lipid parameters were subsequently evaluated in separate multivariable models. In Model 1, HDL-C remained associated with a higher SYNTAX score after adjustment for sex (OR 0.944, 95% CI 0.908–0.980; *p* = 0.003). In Model 2, the TG/HDL-C ratio was also associated with a higher SYNTAX score after adjustment for sex (OR 1.085, 95% CI 1.003–1.175; *p* = 0.041) (Table 3).

### 3.4. ROC Curve and Diagnostic Performance

Receiver operating characteristic (ROC) curve analysis demonstrated that both HDL-C and the TG/HDL-C ratio were significantly associated with a higher SYNTAX score. HDL-C showed a numerically higher discriminatory performance than the TG/HDL-C ratio, with an AUC of 0.659 (95% CI 0.572–0.747, *p* = 0.001) compared with 0.621 (95% CI 0.531–0.711, *p* = 0.014), respectively. However, direct comparison of the two ROC curves using the DeLong test demonstrated that the difference in AUCs was not statistically significant (*p* = 0.268).

**Figure 1:**
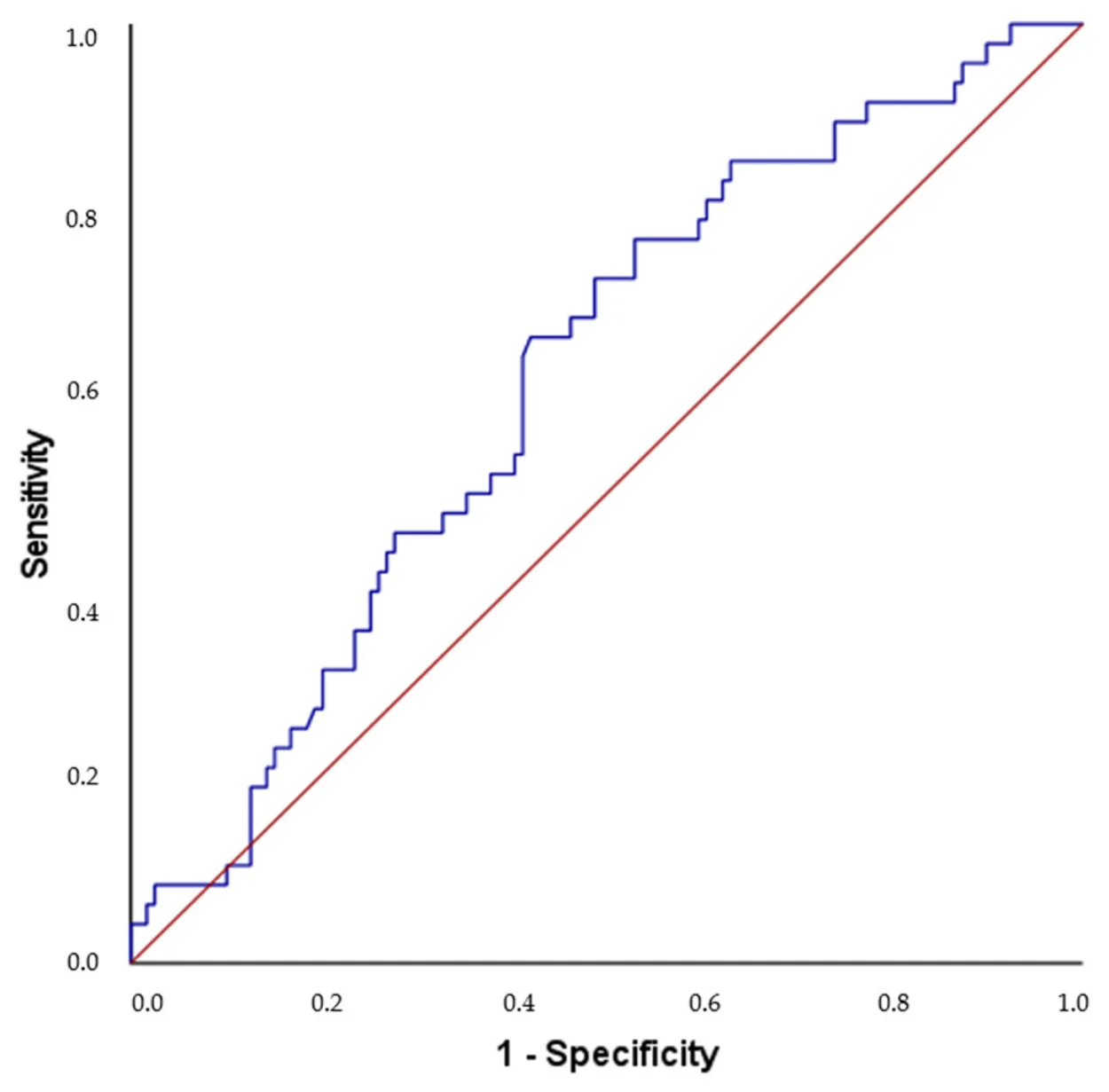
ROC Curve of the TG/HDL-C Ratio for Discriminating a Higher SYNTAX Score. The red diagonal line represents the reference line.

**Figure 2:**
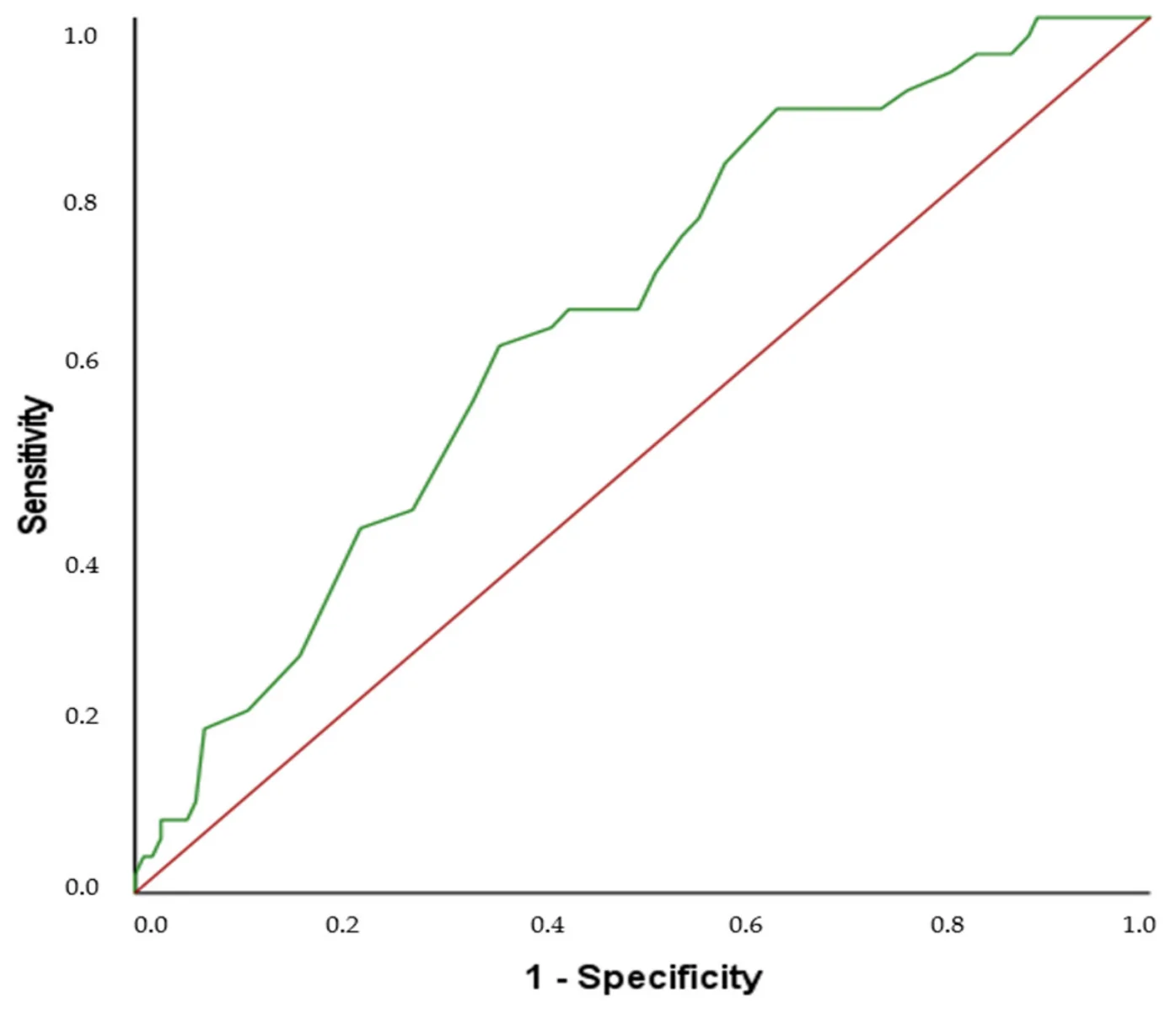
ROC Curve of HDL-C for Discriminating a Higher SYNTAX Score. The red diagonal line represents the reference line.

The optimal HDL-C cut-off value was 36.5 mg/dL, yielding a sensitivity of 63% and a specificity of 64%. For the TG/HDL-C ratio, the optimal cut-off value was 3.98, corresponding to a sensitivity of 67% and a specificity of 58% (Figure 1 and Figure 2 and Table 4).

## 4. Discussion

This study demonstrated that both HDL-C and the TG/HDL-C ratio were associated with angiographic CAD complexity in patients with T2DM. When evaluated in separate multivariable models, both markers remained associated with a higher SYNTAX score after adjustment for sex. Although HDL-C showed a numerically higher AUC, the difference in discriminatory performance between the two markers was not statistically significant. Thus, our findings do not demonstrate a clear superiority of HDL-C over the TG/HDL-C ratio.

The association between HDL-C and coronary complexity is biologically plausible given the atheroprotective functions of HDL. In T2DM, glycation and oxidative stress may impair HDL functionality; therefore, reduced HDL-C may reflect both a quantitative lipid abnormality and dysfunctional HDL associated with vascular injury [2,4,5].

Sex-related differences should also be considered when interpreting HDL-C levels. Women generally have higher HDL-C concentrations than men [17], as also observed in our cohort, while diabetes is associated with a disproportionately greater increase in coronary heart disease risk in women [18]. Nevertheless, HDL-C levels were lower in the higher SYNTAX score group in both sexes and remained associated with a higher SYNTAX score after adjustment for sex.

Population-specific differences in HDL-C levels should also be considered. Previous studies have reported relatively low HDL-C concentrations in Turkish adults compared with several Western populations. This population-specific lipid profile may have influenced the absolute HDL-C values observed in our cohort and should be considered when extrapolating our findings to other populations [19].

The TG/HDL-C ratio reflects insulin resistance and atherogenic dyslipidemia, although its relationship with coronary disease has varied across studies [6,7,20,21]. Its interpretation may also be influenced by sex, as sex-specific thresholds for increased cardiometabolic risk have been proposed and appear to vary across populations [22,23]. However, these thresholds were derived primarily for cardiometabolic risk assessment rather than angiographic CAD complexity. In our cohort, the TG/HDL-C ratio was numerically higher in the higher SYNTAX score group in both women and men, although the sex-stratified differences did not reach statistical significance. Previous epidemiological studies have also demonstrated an inverse association between HDL-C and cardiovascular risk [24,25,26], while heterogeneous findings have been reported for the TG/HDL-C ratio [20,21].

From a clinical perspective, HDL-C is inexpensive and routinely available. Although LDL-C remains the primary therapeutic target, HDL-C may provide complementary information regarding coronary atherosclerotic burden. Low HDL-C should be regarded as a risk marker rather than a direct therapeutic target, as pharmacological strategies aimed at raising HDL-C have not consistently improved cardiovascular outcomes [4,15,27,28,29,30].

Several limitations should be acknowledged. This was a retrospective, single-center study with a relatively modest sample size, limiting causal inference and external validity. HDL functionality, apolipoprotein concentrations, inflammatory biomarkers, and advanced lipoprotein subfractions were unavailable. Detailed information regarding specific lipid-lowering agents, treatment intensity, and concomitant cardiometabolic therapies was not consistently available; therefore, residual confounding related to background pharmacological treatment cannot be excluded. Lipid parameters were obtained from a single fasting sample, and the greater day-to-day biological variability of triglycerides compared with HDL-C may have affected the reproducibility of the TG/HDL-C ratio. Long-term clinical outcomes were also not evaluated. Future prospective multicenter studies incorporating detailed treatment data and clinical outcomes are warranted.

Despite these limitations, strengths of the study include its exclusive focus on patients with T2DM, assessment of coronary complexity using the validated SYNTAX score, formal assessment of multicollinearity, and separate multivariable evaluation of HDL-C and the TG/HDL-C ratio.

## 5. Conclusions

Lower HDL-C and a higher TG/HDL-C ratio remained associated with a higher SYNTAX score after adjustment for sex when evaluated in separate multivariable models. Although HDL-C showed numerically better discriminatory performance, the difference between the two markers was not statistically significant. These routinely available lipid parameters may provide complementary information regarding coronary anatomical complexity in patients with T2DM.

## Figures and Tables

**Table 1:** Baseline Clinical and Laboratory Characteristics According to SYNTAX Score Category.

Variable	Low SYNTAX Score Group (≤22) (*n* = 119)	Higher SYNTAX Score Group (>22) (*n* = 48)	*p*-Value
Age (years)	62.59 ± 10.1	61.60 ± 9	0.371
Body Mass Index (kg/m^2^)	29.16 ± 4.5	30.14 ± 4.8	0.288
Body Surface Area (m^2^)	1.91 ± 0.23	1.97 ± 0.15	0.137
SYNTAX Score	7.14 ± 6.9	30.85 ± 6.2	<0.001
Male Sex, *n* (%)	68 (57.1%)	33 (68.8%)	0.17
Hypertension, *n* (%)	85 (71.4%)	35 (72.9%)	0.85
Atrial Fibrillation, *n* (%)	15 (12.6%)	3 (6.3%)	0.23
Family History, *n* (%)	54 (45.4%)	22 (45.8%)	0.96
Smoking, *n* (%)	42 (35.3%)	23 (47.9%)	0.13
Chronic Kidney Disease, *n* (%)	11 (9.2%)	4 (8.3%)	0.85
Cerebrovascular Disease, *n* (%)	4 (3.4%)	3 (6.3%)	0.39
Triglyceride-to-HDL Cholesterol Ratio	4.63 ± 3.5	6.27 ± 5.8	0.014
Women	4.58 ± 3.4	6.81 ± 5.7	0.059
Men	4.67 ± 3.7	6.02 ± 5.9	0.100
Hemoglobin A1c (%)	7.49 ± 2.2	7.81 ± 1.9	0.162
Serum Creatinine (mg/dL)	1.11 ± 1.2	1.05 ± 0.5	0.111
Hemoglobin (g/dL)	12.66 ± 1.7	12.67 ± 1.9	0.882
HDL Cholesterol (mg/dL) overall	42.88 ± 11.8	36.44 ± 8.7	0.001
Women	46.74 ± 12.1	39.47 ± 9.53	0.037
Men	40 ± 10.81	35.06 ± 8.08	0.022
LDL Cholesterol (mg/dL)	111.75 ± 39.7	107.21 ± 46.9	0.384
Total Cholesterol (mg/dL)	186.11 ± 48.5	177.46 ± 55.3	0.298
Triglycerides (mg/dL)	176.97 ± 98.8	202.15 ± 136.4	0.167

**Table 2:** Correlation Analysis of SYNTAX Score With Clinical and Laboratory Parameters.

Variable	r	*p*-Value
Triglyceride-to-HDL Cholesterol Ratio	0.159	0.040
HDL Cholesterol	−0.339	<0.001
LDL Cholesterol	−0.145	0.063
Triglyceride	0.055	0.485
Age	−0.016	0.835
Body Mass Index	0.104	0.181
Body Surface Area	0.150	0.053
Hemoglobin A1c	0.119	0.136
Creatinine	−0.018	0.818
Hemoglobin	−0.037	0.634

**Table 3:** Univariable and Multivariable Logistic Regression Analyses of Factors Associated with a Higher SYNTAX Score.

Variable	Univariate			Model 1			Model 2		
	OR	95% CI	*p*-Value	OR	95% CI	*p*-Value	OR	95% CI	*p*-Value
Age	0.990	0.956–1.024	0.555						
Male Sex	1.650	0.811–3.357	0.167	1.181	0.554–2.520	0.667	1.674	0.814–3.441	0.161
Body Mass Index	1.047	0.974–1.126	0.212						
Triglyceride-to-HDL Cholesterol Ratio	1.084	1.003–1.171	**0.042**				1.085	1.003–1.175	**0.041**
HDL Cholesterol	0.942	0.907–0.977	**0.001**	0.944	0.908–0.980	**0.003**			
Triglycerides	1.002	0.999–1.005	0.194						
LDL Cholesterol	0.997	0.989–1.006	0.525						
Hemoglobin A1c	1.072	0.917–1.252	0.381						
Creatinine	0.950	0.670–1.347	0.772						
Hemoglobin	1.004	0.829–1.216	0.969						

**Table 4:** ROC Analysis for Discriminating a Higher SYNTAX Score.

Variable	AUC (95% CI)	Cut-Off Value	Sensitivity (%)	Specificity (%)	*p*-Value
Triglyceride-to-HDL Cholesterol Ratio	0.621 (0.531–0.711)	3.98	67	58	0.014
HDL Cholesterol	0.659 (0.572–0.747)	36.5	63	64	0.001

The difference between the AUCs of HDL-C and the TG/HDL-C ratio was not statistically significant (DeLong *p* = 0.268).

## Data Availability

The data presented in this study are available on request from the corresponding author. The data are not publicly available due to ethical reasons.

## Abbreviations

The following abbreviations are used in this manuscript:

CAD	Coronary artery disease
HDL-C	High-density lipoprotein cholesterol
LDL-C	Low-density lipoprotein cholesterol
SYNTAX	Synergy Between Percutaneous Coronary Intervention with TAXUS and Cardiac Surgery
T2DM	Type 2 diabetes mellitus
TG/HDL-C	Triglyceride-to-high-density lipoprotein cholesterol ratio
